# Effects of non-initial radiation exposure on solid cancer mortality risk among Hiroshima A-bomb survivors

**DOI:** 10.3389/fpubh.2025.1651887

**Published:** 2025-09-10

**Authors:** Megu Ohtaki, Keiko Otani, Masaharu Hoshi, Hiroshi Yasuda

**Affiliations:** ^1^The Center for Peace, Hiroshima University, Hiroshima, Japan; ^2^Department of Radiation Biophysics, Research Institute for Radiation Biology and Medicine, Hiroshima University, Hiroshima, Japan

**Keywords:** atomic bomb survivor, non-initial radiation, residual radiation, solid cancer mortality, wind effect, black rain

## Abstract

**Purpose:**

Exposure of atomic bomb (A-bomb) survivors to non-initial (residual) radiation and consequent health effects has not yet been reliably estimated. This study aimed to quantify the contribution of non-initial radiation to the increase in solid cancer mortality risk among A-bomb survivors in Hiroshima through a comparative analysis considering geographical factors.

**Data:**

We analyzed the data of 43,056 (17,603 men and 25,453 women) A-bomb survivors registered in the A-bomb Survivor Cohort Database (ABS) at Hiroshima University. These subjects were aged <50 years old at the time of the bombing and lived in Hiroshima Prefecture as of 1 January 1970, after being exposed within 5.0 km of the hypocenter.

**Methods:**

The radiation doses and excess deaths from all solid cancers of the A-bomb survivors were estimated for districts geographically divided by distance and direction from the hypocenter. The dose was defined as the sum of the initial and non-initial radiation doses, and district-averaged non-initial doses were calculated. The excess relative risks (ERRs) of all solid cancer deaths were estimated using multivariate survival analysis with an additive parametric hazard model under the linear no-threshold (LNT) hypothesis. The γ-ray equivalent doses (Sv) from non-initial radiation were estimated based on the estimated ERRs.

**Results:**

Estimated ERRs were notably higher west of the hypocenter than in the other directions. This trend increased with increasing distance from the hypocenter, and the ERRs in men were higher than those in women. Significantly higher ERR values of 52% (*p* < 0.01) for men and 29% (*p* < 0.05) for women were obtained at a distance of 2.0–2.5 km west of the hypocenter. The γ-ray equivalent doses estimated from these ERRs exceeded 2 Sv of the effective dose in men west of the hypocenter. This level was notably higher than the estimated initial radiation dose.

**Conclusion:**

The findings of this study highlight the considerable contribution of non-initial radiation to the health consequences of the A-bomb survivors. These effects are attributable to the radionuclides generated by the A-bomb detonation, which were assumed to be carried by the wind to the west and deposited with rain in the western region from the hypocenter.

## Introduction

Three large cohort studies on the health effects of atomic bomb survivors have been conducted independently by the Radiation Effects Research Foundation (RERF) (1–3), Hiroshima University (4, 5), and Nagasaki University (6). In many of these studies, chronic disease risks as mortality and incidence, were examined in relation to doses from the atomic bomb (called hereafter “A-bomb”), based on a linear no-threshold (LNT) model (7) or modified models. In the RERF studies, so-called “Life Span Study (LSS),” radiation doses of the A-bomb survivors were calculated using a dosimetry system, known as DS86 (8) or DS02 (9). These dosimetry systems provide only the initial radiation produced directly by the A-bomb detonation, but do not involve the non-initial (residual) radiation from the neutron-activated radionuclides in the surrounding materials and fallout particles. One of the reasons for this omission in the LSS was that the integrated dose from non-initial radiation was estimated to be 20–30 mGy in Hiroshima (10), which was much smaller than the initial radiation dose.

However, soon after the atomic bombing, clinicians and researchers focused their attention on the occurrence of acute radiation sickness (called hereafter “acute symptoms”) among A-bomb survivors, which could not be explained solely by the initial radiation dose (11, 12). Although it is highly difficult to accurately estimate the doses from radioactive microparticles immediately after the A-bomb detonation because reliable relevant data are lacking (13), few studies have attempted to determine the non-initial radiation doses. For example, Oho, a town doctor in Hiroshima, became suspicious of the possible existence of health effects of non-initial radiation during his practice, and conducted a 6-month health study of A-bomb survivors beginning in 1957 (14). He interviewed 3,946 A-bomb survivors and 692 entrants (those who entered the affected area near the hypocenter immediately after the A-bomb detonation in Hiroshima) and asked them about their exposure status, the presence and extent of acute radiation sickness, and behavior during the first 3 months after the bombing. The results showed that entrants had a higher incidence of acute symptoms than those who did not enter the affected area, and this tendency became more pronounced at a closer distance from the hypocenter (14–16). Matsuura et al. used Cox regression with a proportional hazards model to examine the risk of death from malignant neoplasms among early entrants, and found that only survivors who entered the affected area on the day of the atomic bombing (6 August 1945) had a significantly higher risk of death than the control group who entered on 9 August or later (17). Kamada et al. reported that the risk of leukemia among A-bomb survivors during 1970–1990 was 3.7 times (*p* < 0.05) higher for both sexes when the entry date was 6 August than among Japanese individuals in the same period (18). Otani et al. analyzed all solid cancer mortality among survivors who entered Hiroshima early by sex and age group at the time of the bombing (19), based on the multistage carcinogenesis hypothesis (20, 21). They considered the date of entry as a surrogate variable for the non-initial radiation dose and incorporated it into the model of excess relative risk (ERR) for comparison with the mortality risk of controls who entered Hiroshima City after 9 August. As a result, solid cancer mortality risks were significantly higher among persons who entered the City on the day of the bombing than among those who entered the City three or more days later. In addition, it was assumed that middle-aged people who entered the city on the day of the bombing were exposed to higher levels of non-initial radiation than younger people. A possible reason for this trend is presumed to be more engagement of middle-aged people in rescue and search activities for a longer period than other age groups immediately after the A-bomb detonation. Related to this issue, Sawada proposed that exposure to radioactive fallout can be largely attributed to the acute symptoms observed in A-bomb survivors (22).

As another subject related to non-initial radiation exposure, the geographical asymmetrical skew in cancer mortality risk is also difficult to explain in connection with the initial radiation dose alone. This non-circular symmetry in cancer mortality risk was identified through the Cox regression analysis of the LSS data (23). Tonda et al. conducted a semiparametric analysis of solid cancer deaths among A-bomb survivors in Hiroshima and visualized the non-circular symmetry of the mortality risk around the hypocenter (24).

Following these relevant findings and the knowledge that radioactive microparticles produced by the A-bomb in Hiroshima were carried by wind and rain soon after the bombing (25–28), we attempt in this study to conduct a more comprehensive analysis of the health effects attributed to non-initial radiation exposure, for which the distance and direction from the hypocenter can be critical factors.

## Data and methods

### Attributes of the subjects analyzed

In this study, we used data from the A-bomb Survivors Cohort Database, known as ABS, which was developed and managed by the Research Institute for Radiation Biology and Medicine (RIRBM), Hiroshima University (4, 5). The data registered in the ABS were issued by the Atomic Bomb Health Certificate from Hiroshima City or Hiroshima Prefecture. The subjects targeted in this study were A-bomb survivors who lived in the affected area in Hiroshima City at the time of the bombing and were confirmed to be Hiroshima residents on 1 January 1970. Therefore, those who temporarily stayed in Hiroshima City on 6 August 1945, and those who entered the affected area after the bombing were excluded. On the other hand, the A-bomb survivors who moved out of Hiroshima Prefecture after 1 January 1970 and remained contactable were included. The number of subjects fitting for the purpose was 43,056 (17,603 men and 25,453 women), who were in Hiroshima Prefecture as of 1 January 1970 (the year when their first interviews were conducted), aged < 50 years old at the A-bomb detonation, and received the initial radiation exposure within 5.0 km of the hypocenter. The maximum follow-up period was 41 years up to 31 December 2010.

The records of all solid cancer deaths of the people included in the ABS were based on the death certificates prepared by doctors and medical institutions, which were officially collected and organized by the Japanese government (currently the Ministry of Health, Labor and Welfare) as part of the Atomic Bomb Survivors Relief Project, a governmental project for providing relief to A-bomb survivors. Information on these deaths is included in the Vital Statistics Death Schedules released by the Prime Minister's Office. The mortality data of the Hiroshima A-bomb survivors, including the cause of death and migration status (going in or out of Hiroshima City), have been updated on a yearly basis according to the dynamic population statistics provided by Hiroshima City and Hiroshima Prefecture. Deaths due to any causes other than solid cancer were treated as mid-course termination, and those who moved out of Hiroshima Prefecture were excluded. Table 1 summarizes the attributes of these subjects, categorized by sex and age at the time of bombing, and the number of solid cancer deaths for the two age groups who died at < 80 and ≥ 80 years old. To maintain diagnostic accuracy on the death certificate and to avoid the risk of conflicts with other causes of death, the age of death was censored at 80 years in this study.

**Table 1:** Number of male and female subjects who died of solid cancer during the period of 1970 to 2010, classified by age at death and age at the bombing.

**Age at bombing**	**Deaths from all solid cancers**							
	**Men**				**Women**			
	**Subjects**	<**80 y/o**	≥**80 y/o**	**Ratio**^#^ **(%)**	**Subjects**	<**80 y/o**	≥**80 y/o**	**Ratio**^#^ **(%)**
0–9	4,987	321	0	100.0	4,668	217	0	100.0
10–19	5,007	853	57	93.7	5,950	538	62	89.7
20–29	2,125	376	141	72.7	5,721	600	363	62.3
30–39	2,772	466	256	64.5	5,085	481	427	53.0
40–49	2,711	305	223	57.8	4,029	248	26	48.3
All ages	17,602	2,321	677	77.4	25,453	2,084	1,117	65.1

^#^Percentage of the subjects who died of solid cancer before 80 years old.

### Evaluation of radiation dose

Hiroshima University developed a series of dosimetry systems for ABS to assess the health effects of exposure to A-bomb radiation (5). The first version called “ABS93D” (29) was revised to the latest version “ABS16D” by replacing the free-in-air kerma values of initial radiation, following the update of the RERF dosimetry system from DS86 to DS02 (30). In this ABS16D, radiation doses in cases of complicated shielding conditions other than in wooden houses in Japan are treated as missing, and the frequency of such cases is 32% within 2.0 km, although it varies slightly depending on the exposure distance, sex, and age at the time of the A-bomb detonation. Even when radiation doses were calculated according to shielding conditions, there were some discrepancies in the estimated doses due to differences between the ABS and LSS dosimetry systems regarding the methods used to collect information on the location and shielding conditions at the time of exposure. With regard to uncertainty, DS86 assumes that the magnitude of uncertainty for individual doses is generally 30–45%, which increases the risk estimates by 10–15% when such uncertainty is considered (30). In this analysis, we employed a single value of 10 for the relative biological effectiveness (RBE) of neutrons, as used in the original Life Span Study (LSS), and did not analyze separate contributions of neutrons and γ-rays. An upper limit of 4 Gy was set to minimize the effect of anomalous initial dose data on the estimation of dose-response parameters for some individuals exposed near the hypocenter, where the initial dose estimates were unusually high. Similar considerations have been made in recent analyses of cancer mortality and morbidity data in the LSS (1–3).

It should be noted that no system directly measured the non-initial (residual) radiation generated by the A-bombs in Hiroshima and Nagasaki. Therefore, we originally performed a semi-parametric survival regression analysis of the risk of all solid cancer deaths as objective variables, and initial radiation dose and exposure situations (combination of distance and direction from the hypocenter) as explanatory variables, using the data of ABS16D. Such a systematic analysis has not been performed quantitatively in previous studies.

### Locations of survivors at the time of the atomic bombing

**Figure 1:**
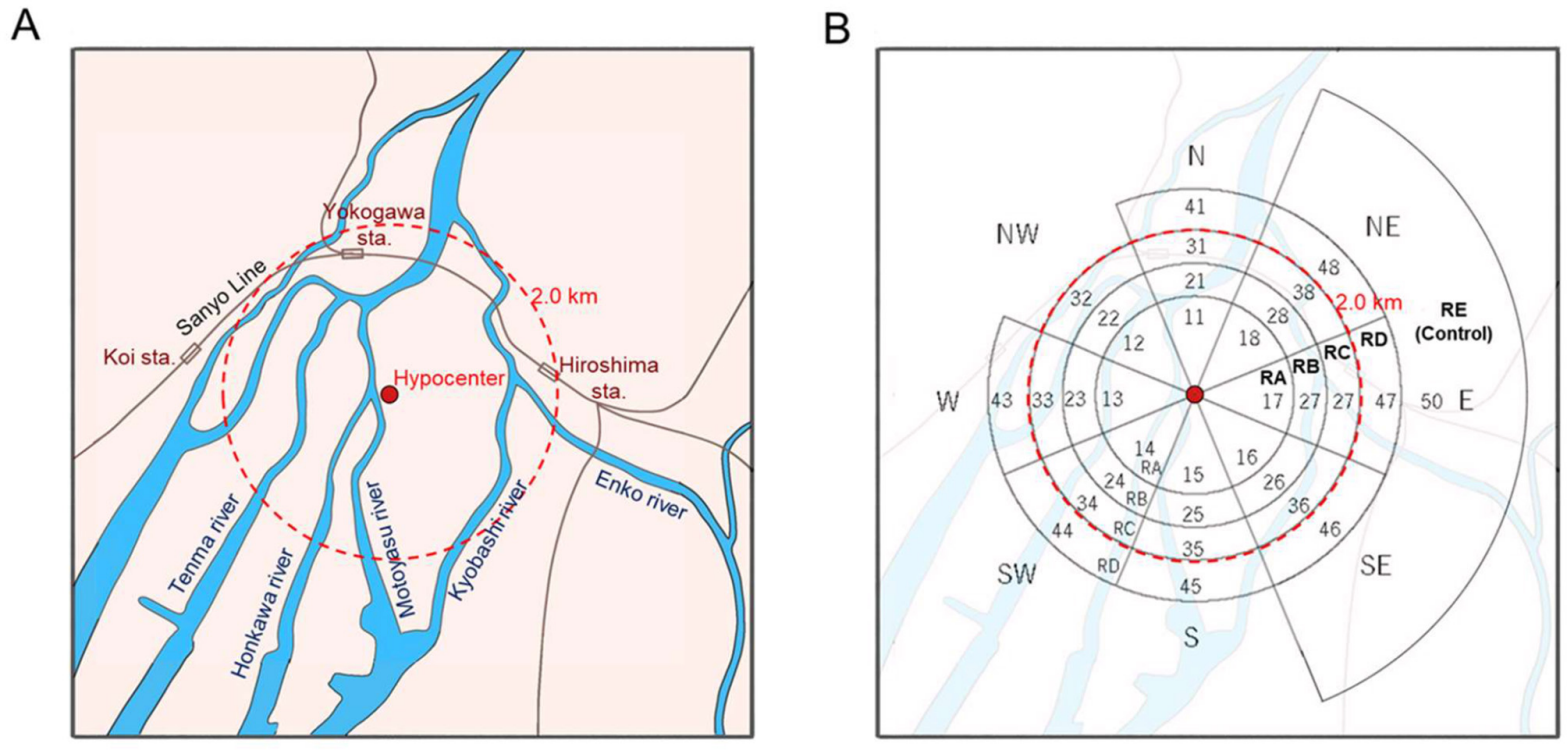
**(A)** A schematic map of the area mainly affected by the Hiroshima atomic bomb, and **(B)** the dividend districts with their codes allocated for the analysis in this study. The code numbers were given to circular arc areas classified by the following distances: less than 1.2 km (RA), between 1.2 km and 1.6 km (RB), between 1.6 km and 2.0 km (RC), between 2.0 km and 2.5 km (RD), and greater than 2.5 km (RE). The RE region was selected as the control.

The area affected by the Hiroshima atomic bomb (Figure 1A) was divided into four ring regions, RA, RB, RC, and RD, in the order of proximity to the hypocenter, and each region was subdivided according to eight azimuthal districts (N, NE, E, SE, S, SW, W, and NW) centered on the hypocenter, as shown in Figure 1B. In addition, circular arc areas located in three directions (northeast, east, and southeast) between 2.5 km and 5.0 km from the hypocenter were added as a control district, resulting in a final set of 33 districts. The rationale for establishing such a control region was that a weak easterly wind blew over the central part of Hiroshima City, including the hypocenter, before and after the A-bomb detonation, and the dust produced by the explosion was carried mainly to the west of the hypocenter (22–25). The location at the time of the bombing can be characterized by the distance and direction from the hypocenter. The following five categories: RA (with the distance less than 1.2 km), RB (between 1.2 km and 1.6 km), RC (between 1.6 km and 2.0 km), RD (between 2.0 km and 2.5 km), and RE (greater than 2.5 km) were established for investigating the location effects of the A-bomb radiation. The RE region was selected as the control region where a hill, named “Hijiyama,” which has an altitude of approximately 100 m above sea level, was expected to have largely prevented non-initial radiation exposure attributed to the A-bomb detonation.

### Overview of the data on solid cancer deaths

Recordings were performed for solid cancer deaths and person-time using sex, age at exposure, attained age, duration of exposure, controls exposed beyond 2.5 km ground distance from hypocenter, DS02R1-weighted colon absorbed dose, and “high dose” index (4.0 Gy as total shielded kerma). The primary outcome was the solid cancer death rate. For reference, person-years of observation (PY) from January 1970 to the earlier date of death, 80th birthday, or December 31, 2010, were also obtained. The exposure distance on the ground from the hypocenter to the location of exposure, direction of the hypocenter, and initial radiation dose (Gy) were treated as exposure status factors, and sex and age at exposure were treated as exposure modifying factors. The relevant data are summarized in Tables 2, 3.

**Table 2:** Number of people by sex, person-years (PY), and solid cancer deaths categorized by district and age.

**District code^#^**	**Age at bombing**	**Men**			**Women**		
		**People**	**PY**	**Deaths**	**People**	**PY**	**Deaths**
RA	0–9	286	8,393	30	296	8,495	33
RA	10–19	421	11,798	76	703	21,335	110
RA	20–29	281	6,533	59	599	15,736	94
RA	30–39	351	5,687	65	429	7,587	61
RA	40–49	373	3,520	44	353	3,655	20
RB	0–9	1,108	32,485	71	1,006	31,197	48
RB	10–19	945	27,527	166	1,122	36,288	94
RB	20–29	401	9,328	73	1,303	34,897	137
RB	30–39	596	9,963	100	1,346	25,434	123
RB	40–49	633	5,849	66	1,123	11,736	74
RC	0–9	1,146	34,437	82	1,114	31,614	43
RC	10–19	1,405	42,165	227	1,780	57,353	140
RC	20–29	602	14,362	102	1,462	40,385	155
RC	30–39	776	12,878	128	1,255	23,147	123
RC	40–49	724	6,760	101	985	10,451	59
RD	0–9	1,302	38,954	79	1,242	37,881	59
RD	10–19	1,422	42,780	255	1,484	47,933	121
RD	20–29	630	15,246	97	1,534	42,333	145
RD	30–39	777	13,223	125	1,353	25,588	114
RD	40–49	726	6,837	63	1,055	11,090	69
RE	0–9	1,145	34,406	59	1,010	30,773	34
RE	10–19	814	24,354	129	861	27,450	73
RE	20–29	211	5,286	45	823	22,434	69
RE	30–39	272	4,516	48	702	13,215	60
RE	40–49	255	5,226	31	513	5,354	26
Total		17,602	419,813	2,321	25,453	625,461	2,084

^#^To ensure that individuals belonging to the RE region were treated as controls, those who entered the city of Hiroshima during the period up to 3 days after the atomic bombing were excluded from analysis.

**Table 3:** Number of people by sex, person-years (PY), and solid cancer deaths categorized by initial radiation level.

**District code**	**Dose (Gy)**	**Men**			**Women**		
		**People**	**PY**	**Deaths**	**People**	**PY**	**Deaths**
RA	0.5 to 1	109	2,342	26	259	5,740	21
RA	1 to 2	425	9,698	61	752	17,565	94
RA	≥2	390	8,567	82	541	12,608	102
RB	0.1 to 0.25	866	20,045	104	1,612	37,074	128
RB	0.25 to 0.5	1,192	28,732	161	2,149	51,155	167
RB	0.5 to 1.0	656	15,762	71	1,120	26,916	99
RB	1.0 to 2.0	41	960	4	34	880	2
RC	0.01 to 0.1	1,942	47,321	292	3,005	75,318	271
RC	0.1 to 0.25	700	16,815	85	1,135	27,647	66
RC	0.25 to 0.5	13	344	0	10	261	2
RD	0.001 to 0.1	1,700	42,410	209	2,472	62,022	181
RE	< 0.001	1,840	49,288	197	2,804	71,172	175
Total		9,874	242,284	1,292	15,893	388,358	1,308

To provide an overview of the geographical distribution of the risk of solid cancer mortality by location at the time of the atomic bombing, district-specific standardized mortality ratios (SMRs) were obtained, with the direction defined by the distance from the hypocenter. For each ring region, the observed number and expected number of deaths were calculated based on the data of all Japanese as the reference population (31) for 41 years from 1970 to 2010 for each age by sex, and the SMRs were calculated, as presented in **Table A1** in Appendix.

### Survival analysis for cancer mortality with a single point additional exposure

In the cohort treated in this study, the hazard function for solid cancer mortality in those of sex s (1: men, 2: women) exposed to initial radiation dose *D* and non-initial radiation dose *R* at age *a* at the time of exposure is assumed to be represented by a linear dose-response model based on the multistage hypothesis of radiation carcinogenesis as follows (1, 2),


(1) $$\begin{array}{l} h\left(t|s,a,D,R\right)=e^{\psi_{s}}\cdot h_{0}\left(t|s,a\right)\left\{1+ERR\left(D,R|s,a,t\right)\right\} \end{array}$$

where $e^{\psi_{s\;}}$ is a correction term that considers the beneficial health effects of the certified A-bomb survivors who have the A-bomb Survivors' Health Handbook issued by the Japanese government, and *h*_0_(*t*|*s, a*) is a spline function that represents the baseline for the solid cancer mortality rate for the entire country of Japan. The ERR of solid cancer deaths was assumed to be represented by a modification of the radiation effect by *a* and attained age *t*, using the following multiplicative log-linear model,


(2) $$\begin{array}{l} ERR\left(D,R|s,a,t\right)=e^{-\tau_{s}\cdot \varphi \left(a\right)-log\left(t/70\right)}\left(\beta_{s}D+R\right) \end{array}$$

where τ_*s*_ is a parameter that expresses the dependence of sensitivity to radiation exposure on age-at-exposure, and φ(*a*) = (*a* − 30/10). As any analysis in this study was conducted separately for each sex, sex-related notations in the relevant equations were omitted. For individuals followed from the starting age to the ending age at a time point higher than the age at exposure, the cumulative hazard can be expressed from Equations 1, 2 as follows:


(3) $$\begin{array}{l} \int_{u}^{v}h\left(t|a,D,R\right)dt=e^{\psi }\int_{u}^{v}h_{0}\left(t|a\right)\{1+e^{-\tau \cdot \varphi \left(a\right)-log\left(t/70\right)}(\beta D\\ \;+R)\}dt \end{array}$$

The distribution of non-initial radiation dose was considered to be a composite of a factor *R*^(*R*)^ that depends only on the exposure distance and a factor *R*^(*W*)^ with east-west deviation due to the east wind effect. Thus, it was assumed that *R* = *R*^(*R*)^+*R*^(*W*)^,


(4) $$\begin{array}{l} R^{\left(R\right)}=R^{\left(R\right)}\left(x,y\right)\equiv R^{\left(R\right)}\left(gdist\right)\;=\;\theta_{A}^{\left(R\right)}I_{A}+\theta_{B}^{\left(R\right)}I_{B}+\theta_{C}^{\left(R\right)}I_{C}\\ \;+\theta_{D}^{\left(R\right)}I_{D} \end{array}$$

(5) $$=\{\begin{array}{cc} \theta_{A}^{\left(R\right)}, & gdist=\sqrt{x^{2}+y^{2}}<1.2km,\;\\ \theta_{B}^{\left(R\right)}, & 1.2km\leq gdist<1.6km,\;\\ \theta_{C}^{\left(R\right)}, & 1.6km\leq gdist<2.0km,\;\\ \theta_{D}^{\left(R\right)}, & 2.0km\leq gdist<2.5km,\\ \;0, & else, \end{array}\;$$

(6) $$\begin{array}{l} R^{\left(W\right)}=\left(\theta_{A}^{\left(W\right)}I_{A}+\theta_{B}^{\left(W\right)}I_{B}+\theta_{C}^{\left(W\right)}I_{C}+\theta_{D}^{\left(W\right)}I_{D}\right)\cdot \frac{\left(-x\right)}{\sqrt{x^{2}+y^{2}}} \end{array}$$

where $\theta_{A}^{\left(R\right)},\ldots ,\theta_{D}^{\left(R\right)},\theta_{A}^{\left(W\right)},\ldots ,\theta_{D}^{\left(W\right)}$ are the parameters to be estimated. These values cannot be directly obtained through measurements, but can be formulated as surrogate quantities using indicator functions of exposure distance, which are denoted by indicator variables *I*_*A*_, …, *I*_*D*_ and the axis of the exposed location (*x, y*) with


(7) $$\begin{array}{l} J_{A}=I_{A}\cdot \frac{\left(-x\right)}{\sqrt{x^{2}+y^{2}}},\ldots ,\;J_{D}=I_{D}\cdot \frac{\left(-x\right)}{\sqrt{x^{2}+y^{2}}}. \end{array}$$

### Logarithmic function of likelihood

The data on deaths from all solid cancers among atomic bomb survivors in Hiroshima from January 1970 to December 2010 were given by (*a*_*i*_, *u*_*i*_, *v*_*i*_, , *D*_*i*_, *R*_*i*_), *i* = 1, …, *n*. If the number of deaths from all solid cancers was the realized value of the random variable indicating the presence or absence of deaths from all solid cancers among A-bomb survivors, the log likelihood is expressed as follows,


(8) $$\begin{array}{l} log\;L\left(\beta ,\theta ,\psi ,\tau \right)=-\sum_{i=1}^{n}\mu_{i}\left(\beta ,\theta ,\psi ,\tau \right)\\ \;+\sum_{i:y_{i}=1}log\left\{h\left(\nu_{i}|\beta ,\theta ,\psi ,\tau \right)\right\} \end{array}$$

where *u*_*i*_ is the age of individual *i* at the beginning of the observation period; *v*_*i*_ is the age at the end of the observation period; and *h*_0_(*t*|*a*) is the hazard of mortality at age *t* for a person whose age was *a* in August 1945 in the reference population (Japanese national average). The expected number (rate) of deaths of individual *i* during the entire observation period is given by


(9) $$\begin{array}{l} \mu_{i}\left(\beta ,\psi ,\tau \right)=e^{\psi }\int_{u_{i}}^{v_{i}}h_{0}\left(t|a_{i}\right)\{1+e^{-\tau \cdot \varphi \left(a_{i}\right)-log\left(t/70\right)}(\beta D_{i}\\ \;+R_{i})\}dt. \end{array}$$

The second term of Equation 8 is expressed as follows,


(10) $$\begin{array}{l} \underset{i:y_{i}=1}{\sum }log\;h\left(v_{i}|\beta ,\theta ,\psi ,\tau \right)=m_{1}\psi +\underset{i:y_{i}=1}{\sum }log\{1+70\\ \;\times e^{-\tau \cdot \varphi \left(a_{i}\right)}\frac{\beta D_{i}+R_{i}}{v_{i}}\}\\ +\sum_{i:y_{i}=1}log\;h_{0}\left(v_{i}|a_{i}\right), \end{array}$$

where *m*_1_ = #{*i*|*y*_*i*_ = 1}. Therefore, the maximum likelihood estimate of the unknown parameters is given by maximizing the following function:


(11) $$\begin{array}{l} Q\;\left(\beta \theta ,\psi ,\tau \right)=-e^{\psi }\overset{n}{\underset{i=1}{\sum }}\{\;\overset{v_{i}}{\underset{t=u}{\sum }}h_{0}\left(t|a_{i}\right)+70\cdot e^{-\tau \cdot \varphi \left(a_{i}\right)}(\beta D_{i}\\ \;+R_{i})\overset{v_{i}}{\underset{t=u_{i}}{\sum }}t^{-1}h_{0}\left(t|a_{i}\right)\}\;+m_{1}\psi +\sum_{i:y_{i}=1}log\{1\\ \;+70\times e^{-\tau \cdot \varphi \left(a_{i}\right)}\frac{\beta D_{i}+R_{i}}{v_{i}}\}, \end{array}$$

The unknown parameters, ψ, τ, β and θ in Equation 11 were estimated using an algorithm for optimization with the limited memory Broyden–Fletcher–Goldfarb–Shanno method (32). These analyses were performed by using the function “optim” in the statistical computing software “R” ver. 4.4.1 (The R Foundation).

## Results

### SMR of solid cancers

**Figure 2:**
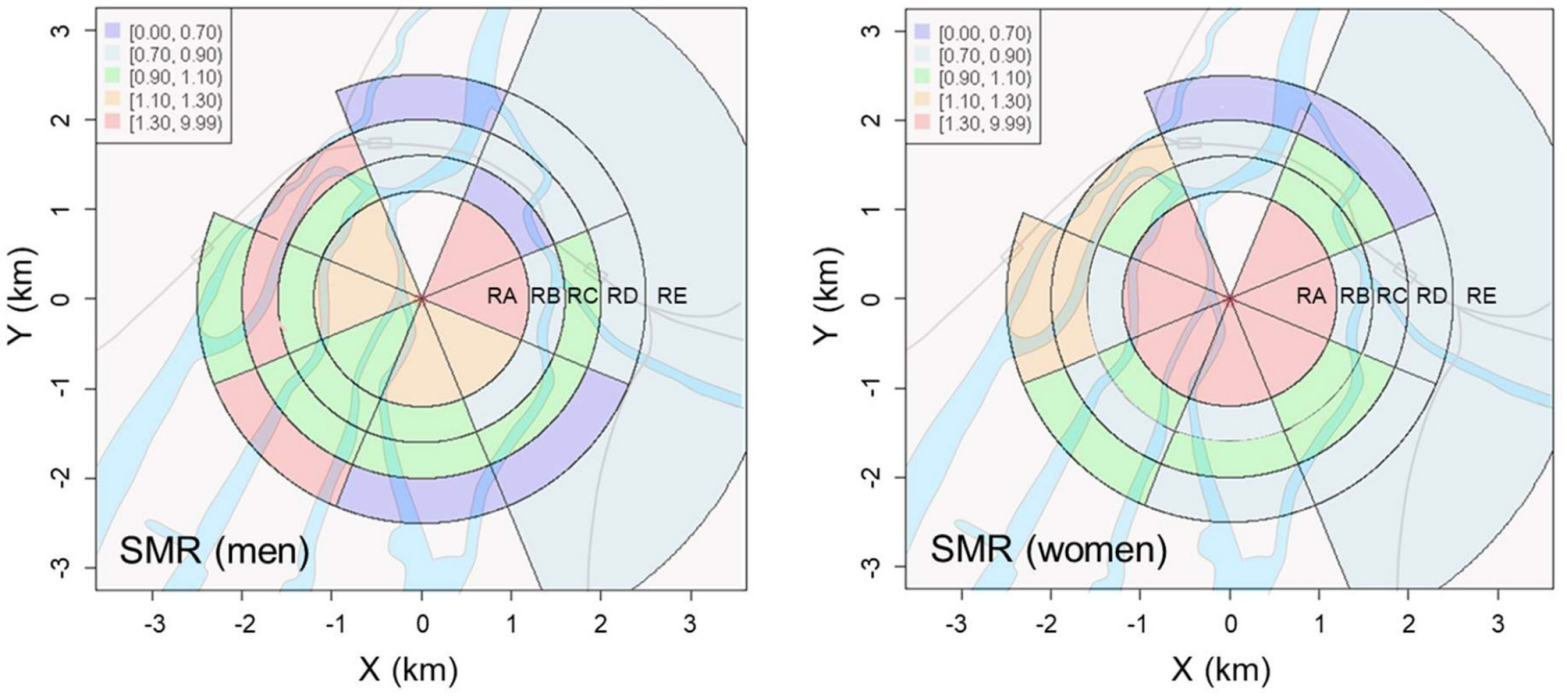
Geographical distributions of standard mortality ratios (SMRs) of solid cancers estimated for men **(Left)** and women **(Right)**.

The geographic distribution of the calculated SMRs for solid cancers in the Hiroshima A-bomb location at the time of bombing is plotted in Figure 2. The SMR values are summarized in Table A1 in Appendix, where the point estimates and 95% confidence intervals for each district's SMR are listed along with the numbers of deaths and expected deaths. The point estimates of SMR for the control (RE) region were 0.828 and 0.838, for men and women, respectively. It was found that the SMR of solid cancer deaths among Hiroshima A-bomb survivors varied with distance from the hypocenter, showing notably different patterns from those of the initial radiation dose. The SMR values did not show a monotonically decreasing trend with distance from the hypocenter, but became higher on the west side of the hypocenter than on the east side, and this tendency was more pronounced in men than in women.

### Goodness of fit of candidate models

Table 4 shows the results of calculating the log likelihood and AIC as indicators of goodness of fit to the data for several models. The table shows that models with ring indicator variables for RB, RC, and RD regions, which represent the distance dependence of excess risk in regions 1.2 km or more from the hypocenter, had a lower goodness of fit than models without these indicator variables. This result suggests that the ground-distance effects of non-initial radiation exposure on the ERR were minor. Nevertheless, the models using ring-direction interaction variables (*J*_*C*_, *J*_*D*_) related to the RC and RD regions for the trends of the east-west difference improved the goodness of fit. Among the candidate models, Model 10 was selected as the optimal model minimizing AIC, which did not use ring area indicators (*I*_*B*_, *I*_*C*_, *I*_*D*_) but used ring-direction interaction variables (*J*_*C*_, *J*_*D*_). Model 12, which used only initial radiation exposure, had the lowest goodness of fit.

**Table 4:** Goodness of fit for candidate models, Likelihood (LLK) and AIC with 12 models.

**Model no**.	**Variable usage** ^**#**^									***k* ^$^**	**Men**		**Women**	
	***I**_β_*	***I**_*A*_*	***I**_*B*_*	***I**_*C*_*	***I**_*D*_*	***J**_*A*_*	***J**_*B*_*	***J**_*C*_*	***J**_*D*_*		**LLK**	**AIC**	**LLK**	**AIC**
1	1^#^	1	1	1	1	1	1	1	1	9	−1347.3	2716.6	−1284.8	2591.7
2	1	1	1	1	0	1	1	1	1	8	−1348.6	2717.2	−1285.0	2590.0
3	1	1	1	0	1	1	1	1	1	8	−1348.3	2716.6	−1284.9	2589.9
4	1	1	0	1	1	1	1	1	1	8	−1348.7	2717.3	−1284.9	2589.7
5	1	1	1	0	0	1	1	1	1	7	−1347.3	2712.7	−1285.2	2588.3
6	1	1	0	1	0	1	1	1	1	7	−1347.4	2712.8	−1285.0	2588.0
7	1	1	0	0	1	1	1	1	1	7	−1347.7	2713.5	−1285.0	2587.9
8	1	1	0	0	0	1	1	1	1	6	−1347.8	2711.6	−1285.3	2586.5
9	1	1	0	0	0	0	1	1	1	5	−1349.3	2712.6	−1285.3	2584.5
**10** ^*^	**1**	**1**	**0**	**0**	**0**	**0**	**0**	**1**	**1**	**4**	**−1349.5**	**2711.0**	**−1285.3**	**2582.5**
11	1	1	0	0	0	0	0	0	1	3	−1358.2	2726.4	−1286.7	2583.4
12	1	0	0	0	0	0	0	0	0	1	−1361.4	2728.9	−1288.9	2583.8

^#^“1” indicates when the variable is used, “0” indicates when the variable is not used in the model.

^$^“k” indicates the number of variables concerning the exposed location in the model.

^*^Model 10 indicated in boldface is considered optimal in terms of minimizing AIC.

### Estimates of parameters for the ERR of solid cancer mortality among A-bomb survivors

The properties of the parameter estimates were inspected based on Model 9, which included the optimal model (Model 10) as one of the closest submodels. Table 5 shows the point estimates of β, θ^(*R*)^, θ^(*W*)^, ψ* and τ*, standard errors, 95% confidence intervals and statistical significance resulting from fitting with Model 9 (see Table 4). For men, the point estimate of the ERR due to non-initial radiation was 0.191 in the RA region adjacent to the hypocenter, which was much higher than 0.120 because of the initial radiation exposure of approximately 1 Gy, although it was not statistically significant. Regarding the west high effect (*R*^(*W*)^), a highly significant excess risk of more than 30% is detected on the west side of the RC and RD regions, with a significance of p < 0.01. For women, except for the west side of the RD region, no location dependence with a statistical significance was detected. Table 6 shows the results of the analysis when only the effect of the initial radiation dose was modeled, which were almost identical to those in the LSS report (1).

**Table 5:** Estimated regression coefficients and relevant data for an optimized model with initial radiation and non-initial radiation exposures.

**Source of dose**	**Men** ^**#**^				**Women** ^**$**^			
	**Coef**.	**S.E**.	**(95% CI)**	***P*** **-value**	**Coef**.	**S.E**.	**(95% CI)**	***P*** **-value**
**Initial radiation effect per Gy (**β**)**								
I_β_	0.12	0.061	(0.000 to 0.240)	0.0504	0.394	0.083	(0.231 to 0.557)	< 0.001
**Non-initial radiation (ring region) effect: [**θ^(R)^**]**								
I_A_	0.191	0.139	(−0.081 to 0.463)	0.1685	0.127	0.138	(−0.143 to 0.395)	0.357
I_B_	0	–	–	–	0	–	–	–
I_C_	0	–	–	–	0	–	–	–
I_D_	0	–	–	–	0	–	–	–
**Non-initial radiation (high deviation in the west) effect: [**θ^(W)^**]**								
J_A_	0	–	–	–	0	–	–	–
J_B_	0.142	0.071	(0.003 to 0.281)	0.0474^*^	−0.043	0.06	(−0.161 to 0.075)	0.4707
J_C_	0.300	0.087	(0.129 to 0.471)	< 0.001	0.154	0.077	(0.003 to 0.305)	0.0462^*^
J_D_	0.521	0.170	(0.188 to 0.854)	0.0021^**^	0.288	0.129	(0.035 to 0.541)	0.0255^*^
Phi	−0.185	0.026	(−0.236 to −0.134)	< 0.001	−0.207	0.029	(−0.264 to −0.150)	< 0.001
Tau	0.164	0.085	(−0.0026 to 0.331)	0.0551	0.337	0.071	(0.198 to 0.476)	< 0.001

^*^p < 0.05, ^**^p < 0.01.

^#^For men, *n* = 9,864, Loglik = −1349.3 (d.f. = 9,857), AIC = 2712.6.

^$^For women, *n* = 15,878, Loglik = −1285.1 (d.f. = 15,871), AIC = 2584.3.

**Table 6:** Estimated regression coefficient and relevant data for the model with only initial radiation exposure.

**Source of dose**	**Men** ^**#**^				**Women** ^**$**^			
	**Coef**.	**S.E**.	**(95% CI)**	***P*** **-value**	**Coef**.	**S.E**.	**(95% CI)**	***P*** **-value**
**Initial radiation effect per Gy (**β**)**								
I_β_	0.140	0.041	(0.060 to 0.220)	< 0.001	0.409	0.056	(0.299 to 0.519)	< 0.001
Phi	−0.123	0.023	(−0.168 to −0.078)	< 0.001	−0.186	0.025	(−0.235 to −0.137)	< 0.001
Tau	0.323	0.151	(0.027 to 0.619)	0.0326^*^	0.353	0.072	(0.212 to 0.494)	< 0.001

^*^p < 0.05.

^#^For men, *n* = 9,864, Loglik = −1361.4 (d.f. = 9,861), AIC = 2728.9.

^$^For women, *n* = 15,878, Loglik = −1288.9 (d.f. = 15,875), AIC = 2583.8.

According to the point estimates of the ERRs of all solid cancer deaths among A-bomb survivors in the control (RE) region, anti-log of phi (*e*^ψ^) was 0.831 for men and 0.813 for women, both of which were significantly lower than 1.0. This implies that A-bomb survivors treated as controls in this analysis had a lower risk of solid cancer death than the Japanese national average general population. When the optimal model was applied, the age-at-exposure-dependent coefficient of radiosensitivity (τ) was estimated as 0.167 for men and 0.337 for women.

**Figure 3:**
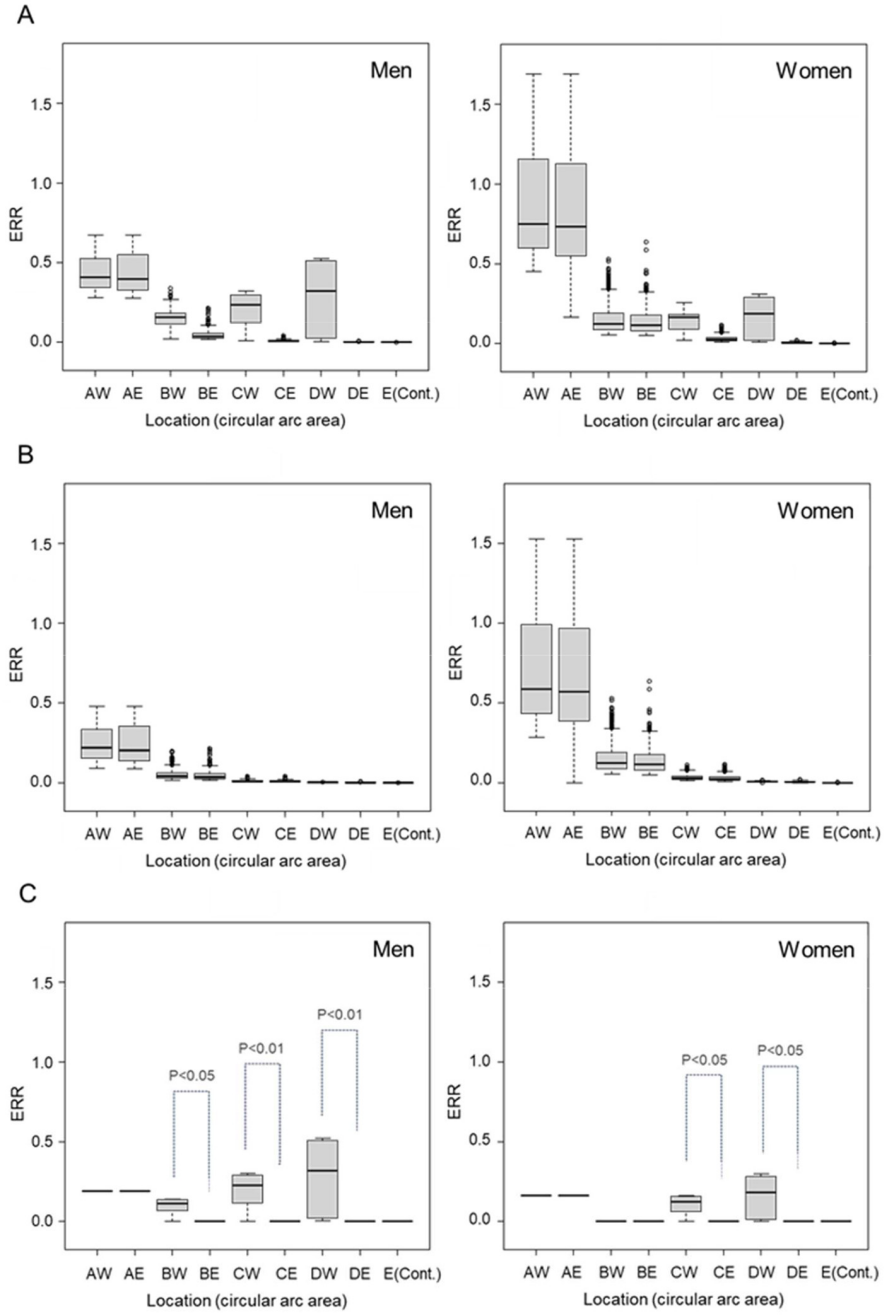
Boxplots of estimated excess relative risks (ERRs) in the circular arc areas categorized by direction from the hypocenter (see Figure 1B) for men (left) and women (right): **(A)** estimated ERRs attributed to total radiation exposures, **(B)** estimated ERRs attributed to initial radiation exposure, and **(C)** estimated ERRs attributed to non-initial (residual) radiation exposure. AW, BW, CW, and DW are the western half areas of the RA, RB, RC, and RD, respectively, and AE, BE, CE, and DE are the eastern half areas of the RA, RB, RC, and RD, respectively.

Parallel boxplots of estimated ERRs for persons of the attained age of 70 years old after exposure at the age of 30 years old, simplifying the calculation of Equation 2, by the western and eastern semi-annular regions and sex are shown in Figure 3. The top panel (A), which presents the parallel boxplots of the ERR distribution due to total (initial and non-initial) radiation, shows a difference between the western and eastern semi-annular area (RB, RC, and RD regions) by sex. The middle panel (B), which presents similar boxplots for initial radiation, shows no difference between the western and eastern semi-annular regions in each common ring region. The bottom panel (C), which presents the ERRs due to non-initial radiation exposure, shows that the western semi-annular area (RC and RD regions) had much higher ERRs than the eastern semi-annular regions. This trend was more pronounced for men, noting that the ERRs for men became higher west of the hypocenter (RC and RD regions) beyond 1.6 km from the hypocenter, and were almost the same at the area adjacent to the hypocenter (RA region).

Based on the district-specific ERRs of all solid cancer deaths attributed to non-initial radiation, we estimated the dose due to non-initial radiation in sieverts by district by calculating the ratio of the increase in ERR due to initial radiation exposure per Gy to the estimated value. For example, the ERR from non-initial radiation within 1.2 km of the hypocenter (RA region) was calculated as 0.191 for men and 0.127 for women. Since the ERR per 1 Gy of initial dose is 0.120 for men and 0.394 for women, the average non-initial dose (in Sv) in the RA region was estimated to be 0.191/0.120 = 1.59 (Sv) for men and 0.127/0.394 = 0.32 (Sv) for women. These non-initial radiation doses include the chronic exposures of the A-bomb survivors registered in the Hiroshima University cohort database (ABS) as of 1970, that is, the accumulated doses for approximately 25 years from August 6, 1945. However, according to the half-lives of the major radionuclides produced by the A-bomb neutrons (33), it is presumed that these survivors received most of the non-initial doses within several days after the A-bomb detonation.

**Figure 4:**
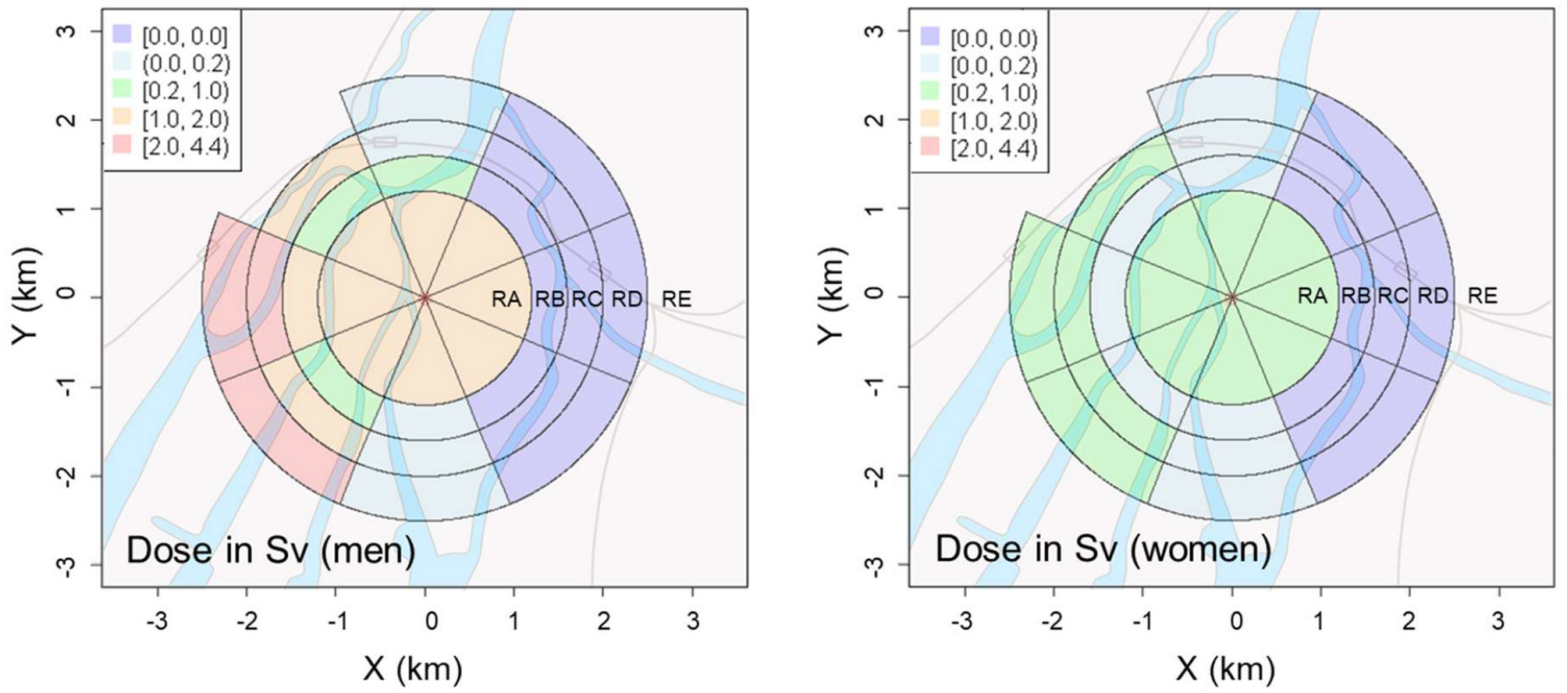
Geographical distributions of radiation dose from non-initial radiation induced by the A-bomb in Hiroshima City for men **(Left)** and women **(Right)**.

The estimated district-specific doses (Sv) attributed to non-initial radiation exposure are shown in Figure 4. Detailed numerical data are summarized in Table A2 in Appendix. For both men and women, while non-initial radiation doses were notably high near the hypocenter (RA region) as expected (34), high doses were similarly obtained west of the hypocenter. Particularly for men, the radiation doses in the area 2 km west of the hypocenter exceeded 2 Sv, which was higher than that near the hypocenter (RA region). In contrast, the estimated doses east of the hypocenter were remarkably low (less than 0.01 Sv) at distances greater than 1.2 km.

## Discussion

### Background of solid cancer risks in Japan

According to estimates from the National Cancer Center Japan (31), the SMR of Hiroshima Prefecture from 2009 to 2013 was 0.971 for men and 0.911 for women. In our analysis using the data of the Japanese population (35) as reference, the SMR of the control (RE) region, where all people were the Health Handbook holders (i.e., certified A-bomb survivors), was 0.831 (*e*^−0.185^) for men and 0.813 (*e*^−0.207^) for women. While the initial and non-initial radiation doses of the A-bomb survivors in the control region were both nearly zero, this observation should not be interpreted simply as hormesis effects (19, 36), because this can be partially explained by the beneficial effects of holding the A-bomb Survivor (Hibakusha) Certificate, which may have outweighed the adverse effects of A-bomb radiation exposure, as these certified survivors were able to receive free medical checkups and treatments that could effectively have reduced the risk of solid cancer death. Future studies should cautiously discuss whether this phenomenon is specific to solid cancer deaths or is observable in cancer incidence and other chronic diseases.

#### Factors contributing to the geographical distribution of ERRs

We found that the risk of solid cancer deaths increased in the western suburbs of the hypocenter for both men and women. Because a weak easterly wind blowing to the west over the city of Hiroshima was observed on the day of the atomic bombing (25, 27), it was probable that radioactive microparticles produced by neutron activation near the hypocenter were carried by the easterly winds to the west of the hypocenter, and caused radioactive contamination due to the rain (known as “black rain”) in these regions.

The point estimates of the ERRs for all solid cancer deaths in the hypocenter area suggest that the contribution of the non-initial radiation dose (Figure 3C) was as large as that of the initial radiation dose (Figure 3B) (11, 12, 36). It should be noted that statistically significant differences were not found (Table 5) owing to the small number of subjects exposed in the vicinity of the hypocenter and the multicollinearity problem caused by the large (negative) correlation between initial dose and distance from the hypocenter. Although the upward deviation of ERR near the hypocenter below 1.2 km may be explained by applying a non-linear (higher-order) dose-response model or by assuming a higher value (>10) of neutron RBE (37–39), the issue regarding the upward deviation of ERR confined to the west of the hypocenter above 1.2 km remains unsolved. This result may be due to the non-negligible effect of secondary radiation from the radionuclides that would have been produced in large quantities owing to the neutron activation.

### Individual differences in non-initial radiation dose

As the non-initial radiation dose is dependent on the time period of exposure, the post-detonation behavior that could affect the exposure time is considered to be an important factor in individual dose assessments. The age and sex (gender) of the exposed person are major factors related to their behavior. Figure 4 and Table A2 in Appendix indicate that the non-initial radiation dose for men was several times higher than that for women in most areas. Thus, it is assumed that men were likely to have remained more active than women soon after the A-bomb detonation through urgent work, such as rescue and searching. Although a more precise analysis considering age at exposure is expected to reveal this association, such a comprehensive analysis is currently difficult owing to insufficient data.

As a rare relevant study, Oho compared the incidence of acute symptoms among those who went to the hypocenter immediately after the A-bomb detonation with those who did not (14). They attempted to explain the status of non-initial radiation exposure and related the observed health effects to immediate post-bombing behaviors. This finding indicates that the health effects of non-initial radiation are dependent on individual behaviors, rather than the distance from the hypocenter, that is, the initial radiation dose.

### Possible source of non-initial radiation

According to a few previous studies regarding the contribution of non-initial radiation, the estimated initial radiation doses to A-bomb survivors who were exposed at distances greater than 2.5 km from the hypocenter or who entered the city were at most several tens of mGy (10, 40, 41). In contrast, the doses estimated in this study (>300 mSv for women and >1.5 Sv for men in the vicinity of the hypocenter, and >400 mSv for women and 2.0 Sv for men in a large area 2 km west of the hypocenter) were significantly higher than the previously reported doses (10, 40, 41). This difference can be explained by the fact that the previous dose estimates did not consider exposure from inhalation of airborne radioactive dust (mostly microparticles), whereas our dose estimates did. The results of this study imply that the contribution of such inhaled radioactive particles to the radiation exposure of A-bomb survivors was notably higher than previously thought, although further research is needed to verify this implication.

Tanaka et al. examined stable chromosome aberration in peripheral blood lymphocytes of 17 crew members of eight fishing vessels and two crew members of one cargo ship in detail by the G banding method 60 years after the nuclear tests conducted by the United States at the “Bravo” hypocenters on Bikini Atoll and Eniwetok Atoll in the Marshall Islands (42). The crew of tuna fishing boats and cargo ships operated approximately 150–1,200 km from the test sites at the time of hydrogen bomb detonation and received exposures to radioactive fallout. Compared to nine age-matched controls, they found that the percentage of stable-type abnormalities was 3.35% in the exposed group, which was significantly higher (by 2.45%) than that in the control group (42). After a comparison of the half-lives of the major radionuclides, ^56^Mn (half-life: 2.6 h) and ^28^Al (half-life: 2.2 min) have emerged as essential sources. Although the effect of ^24^Na (half-life 15.0 h) cannot be ruled out, the results of the ABS-based study by Matsuura et al. (17) and by Otani et al. (19) showed that the excess relative risk for those who entered the city on 6 August was significantly higher than that for those who entered on 9 August, as the dependence of the risk of shape cancer among A-bomb survivors on the date of entry, and those who entered after 10 August, and the excess relative risk for those who entered the market after 10 August was reduced to a few percent. Thus, it is unlikely that exposure to ^24^Na, which has a relatively long half-life of 15 h, brought a significant effect. In the case of the A-bomb detonation in Hiroshima, fine particles containing ^28^Al and ^56^Mn may have caused considerable radiation exposure and the resultant increase of cancer risk, as suggested in previous studies (43, 44). In a recent experimental study on this subject using rats by Hoshi et al., the magnitude of the health effects of hot particles was 20 times higher than that of uniform exposure to the same absorbed dose of gamma-rays in terms of pathology and gene expression (45).

### Limitations of this study

Because the development of this cohort began in 1970, the death records for the period immediately after the bombing (1945–1969) were not included in this study. Accordingly, the causal relationship between mortality and radiation dose may be distorted for cancers with a short latency period, such as thyroid cancer (latency period: 15–20 years), although this effect is considered small, as many radiation-induced solid cancers normally require more than 25 years after exposure to appear. However, we should recognize that various types of biases emerged for 25 years or more after the end of World War II.

Our analysis suggests that the dose rate and duration of non-initial radiation exposure of A-bomb survivors were largely affected by their behavior soon after the bombing, which could greatly vary depending on individual situations. Although the estimated cumulative non-initial radiation dose was reported to be several tens of mGy in previous studies (10, 40, 41), these estimates were likely underestimated because they did not consider possible changes in individual behavior. Unfortunately, it is almost impossible to precisely calculate the individual doses based on their behavior, owing to the chaotic situation immediately after the A-bomb detonation. Further efforts are required to achieve more accurate assessments of the doses and risks of A-bomb radiation on an individual basis.

## Conclusion

In this study, we pointed out that the geographic and sex differences in solid cancer deaths among the A-bomb survivors who lived in the affected area cannot be explained by initial radiation alone, and implied that the non-initial (residual) radiation, which was attributable to radioactive microparticles carried by the east wind and rain, was likely to have contributed to the observed increase in the excess relative risk (ERR) in the western region from the hypocenter. Our analysis indicates the additional health effects of late exposure to non-initial radiation generated by A-bomb detonation. These findings should be useful for more reliable evaluations of the potential risks of nuclear weapons and for discussing effective measures to minimize the medical consequences in a possible nuclear emergency situation in the future. Further efforts are required to precisely determine the dose distribution of non-initial radiation and to explain the dose-dependent excess mortality risk due to a specific cancer on an individual basis. In parallel, it is desirable to further develop relevant epidemiological and animal studies to clarify the biological effects of inhalation and ingestion of A-bomb-induced radioactive microparticles, which are still poorly understood.

## Data Availability

The original contributions presented in the study are included in the article/supplementary material, further inquiries can be directed to the corresponding author.
