# Supplementary material for: Effects of non-initial radiation exposure on solid cancer mortality risk among Hiroshima A-bomb survivors
Source: Front Public Health. 2025 Sep 10;13:1651887. doi: 10.3389/fpubh.2025.1651887 (PMC12457108; doi:10.3389/fpubh.2025.1651887)
Supplement: Supplementary file 1 [file Table_1.docx]

# Appendix

Table A1. District-specific SMR of solid cancer deaths with 95% confidence intervals with the numbers of observed and expected deaths among Hiroshima A-bomb survivors in 1970-2010.

| District code | Distance | Direc-tion | Men | | | | |  | Women | | | | |
| --- | --- | --- | --- | --- | --- | --- | --- | --- | --- | --- | --- | --- | --- |
|  |  |  | Obsvd. deaths | Expct.  deaths | SMR | 95%LCL | 95%UCL |  | Obsvd. deaths | Expct. deaths | SMR | 95%LCL | 95%UCL |
| 11 | <1.2km | N | 0 | 0.66 | 0 | － | － |  | 0 | 0.21 | 0 | － | － |
| 12 | <1.2km | NW | 22 | 18.84 | 1.167 | 0.731 | 1.704 |  | 38 | 21.05 | 1.805 | 1.277 | 2.423 |
| 13 | <1.2km | W | 27 | 21.03 | 1.284 | 0.846 | 1.812 |  | 25 | 15.69 | 1.593 | 1.031 | 2.276 |
| 14 | <1.2km | SW | 19 | 19.24 | 0.988 | 0.594 | 1.479 |  | 38 | 21.43 | 1.774 | 1.255 | 2.38 |
| 15 | <1.2km | S | 18 | 15.84 | 1.137 | 0.673 | 1.719 |  | 30 | 14.92 | 2.011 | 1.356 | 2.792 |
| 16 | <1.2km | SE | 30 | 26 | 1.154 | 0.778 | 1.602 |  | 45 | 26.18 | 1.719 | 1.254 | 2.256 |
| 17 | <1.2km | E | 39 | 24.18 | 1.613 | 1.147 | 2.157 |  | 36 | 22.79 | 1.58 | 1.106 | 2.136 |
| 18 | <1.2km | NE | 14 | 8.3 | 1.688 | 0.922 | 2.68 |  | 5 | 2.91 | 1.715 | 0.553 | 3.514 |
| 21 | [1.2, 1.6km) | N | 27 | 33.87 | 0.797 | 0.525 | 1.125 |  | 52 | 60.81 | 0.855 | 0.639 | 1.103 |
| 22 | [1.2, 1.6km) | NW | 55 | 56.91 | 0.966 | 0.728 | 1.238 |  | 51 | 56.5 | 0.903 | 0.672 | 1.167 |
| 23 | [1.2, 1.6km) | W | 78 | 75.88 | 1.028 | 0.812 | 1.268 |  | 76 | 85.3 | 0.891 | 0.702 | 1.102 |
| 24 | [1.2, 1.6km) | SW | 39 | 39.37 | 0.99 | 0.704 | 1.325 |  | 61 | 56.08 | 1.088 | 0.832 | 1.377 |
| 25 | [1.2, 1.6km) | S | 30 | 33.12 | 0.906 | 0.611 | 1.258 |  | 32 | 42.57 | 0.752 | 0.514 | 1.034 |
| 26 | [1.2, 1.6km) | SE | 53 | 58.98 | 0.899 | 0.673 | 1.156 |  | 78 | 72.63 | 1.074 | 0.849 | 1.325 |
| 27 | [1.2, 1.6km) | E | 45 | 50.22 | 0.896 | 0.653 | 1.176 |  | 63 | 72.85 | 0.865 | 0.664 | 1.091 |
| 28 | [1.2, 1.6km) | NE | 13 | 23.1 | 0.563 | 0.299 | 0.907 |  | 27 | 27.88 | 0.968 | 0.638 | 1.366 |
| 31 | [1.6, 2.0km) | N | 34 | 44.3 | 0.768 | 0.531 | 1.046 |  | 22 | 25.82 | 0.852 | 0.534 | 1.243 |
| 32 | [1.6, 2.1km) | NW | 45 | 31.59 | 1.424 | 1.039 | 1.87 |  | 32 | 26.44 | 1.21 | 0.828 | 1.664 |
| 33 | [1.6, 2.2km) | W | 55 | 39.75 | 1.384 | 1.042 | 1.772 |  | 43 | 37.58 | 1.144 | 0.828 | 1.511 |
| 34 | [1.6, 2.3km) | SW | 39 | 40.43 | 0.965 | 0.686 | 1.29 |  | 29 | 33.98 | 0.853 | 0.571 | 1.191 |
| 35 | [1.6, 2.4km) | S | 38 | 40.62 | 0.936 | 0.662 | 1.256 |  | 40 | 38.56 | 1.037 | 0.741 | 1.383 |
| 36 | [1.6, 2.5km) | SE | 37 | 39.09 | 0.947 | 0.666 | 1.275 |  | 24 | 24.17 | 0.993 | 0.636 | 1.428 |
| 37 | [1.6, 2.6km) | E | 99 | 105.05 | 0.942 | 0.766 | 1.137 |  | 59 | 76.95 | 0.767 | 0.584 | 0.974 |
| 38 | [1.6, 2.7km) | NE | 30 | 42.14 | 0.712 | 0.48 | 0.988 |  | 46 | 43.54 | 1.056 | 0.773 | 1.383 |
| 41 | [2.0, 2.5km) | N | 20 | 29.91 | 0.669 | 0.408 | 0.992 |  | 20 | 29.36 | 0.681 | 0.416 | 1.011 |
| 43 | [2.0, 2.5km) | W | 20 | 18.21 | 1.098 | 0.67 | 1.629 |  | 19 | 16.77 | 1.133 | 0.682 | 1.697 |
| 44 | [2.0, 2.5km) | SW | 40 | 26.95 | 1.484 | 1.06 | 1.978 |  | 25 | 23.37 | 1.07 | 0.692 | 1.528 |
| 45 | [2.0, 2.5km) | S | 17 | 25.85 | 0.658 | 0.383 | 1.005 |  | 21 | 24.08 | 0.872 | 0.54 | 1.283 |
| 46 | [2.0, 2.5km) | SE | 22 | 44.37 | 0.496 | 0.311 | 0.723 |  | 33 | 40.96 | 0.806 | 0.555 | 1.103 |
| 47 | [2.0, 2.5km) | E | 55 | 70.94 | 0.775 | 0.584 | 0.993 |  | 62 | 75.65 | 0.82 | 0.628 | 1.036 |
| 48 | [2.0, 2.5km) | NE | 35 | 40.06 | 0.874 | 0.608 | 1.186 |  | 1 | 4.02 | 0.249 | 0.003 | 0.914 |
| 50 | [2.5, 5.0km) | N~S | 197 | 229.22 | 0.859 | 0.744 | 0.984 |  | 175 | 224.73 | 0.779 | 0.668 | 0.898 |

Table A2. Estimated district-specific mean doses from non-initial radiation exposure attributed to the Hiroshima atomic bomb.

|  |  |  | Dose (Sv) | |
| --- | --- | --- | --- | --- |
| District code | Distance | Direction | Men | Women |
| 11 | <1.2km | N | 1.59 | 0.32 |
| 12 | <1.2km | NW | 1.59 | 0.32 |
| 13 | <1.2km | W | 1.59 | 0.32 |
| 14 | <1.2km | SW | 1.59 | 0.32 |
| 15 | <1.2km | S | 1.59 | 0.32 |
| 16 | <1.2km | SE | 1.59 | 0.32 |
| 17 | <1.2km | E | 1.59 | 0.32 |
| 18 | <1.2km | NE | 1.59 | 0.32 |
| 21 | [1.2, 1.6km) | N | 0.22 | 0.01 |
| 22 | [1.2, 1.6km) | NW | 0.9 | 0.01 |
| 23 | [1.2, 1.6km) | W | 1.15 | 0.02 |
| 24 | [1.2, 1.6km) | SW | 0.82 | 0.04 |
| 25 | [1.2, 1.6km) | S | 0.11 | 0 |
| 26 | [1.2, 1.6km) | SE | 0 | 0 |
| 27 | [1.2, 1.6km) | E | 0 | 0 |
| 28 | [1.2, 1.6km) | NE | 0 | 0 |
| 31 | [1.6, 2.0km) | N | 0.17 | 0.02 |
| 32 | [1.6, 2.0km) | NW | 1.65 | 0.26 |
| 33 | [1.6, 2.0km) | W | 2.45 | 0.38 |
| 34 | [1.6, 2.0km) | SW | 1.74 | 0.28 |
| 35 | [1.6, 2.0km) | S | 0.2 | 0.03 |
| 36 | [1.6, 2.0km) | SE | 0 | 0 |
| 37 | [1.6, 2.0km) | E | 0 | 0 |
| 38 | [1.6, 2.0km) | NE | 0 | 0 |
| 41 | [2.0, 2.5km) | N | 0.07 | 0.01 |
| 43 | [2.0, 2.5km) | W | 4.3 | 0.72 |
| 44 | [2.0, 2.5km) | SW | 2.68 | 0.45 |
| 45 | [2.0, 2.5km) | S | 0.05 | 0.02 |
| 46 | [2.0, 2.5km) | SE | 0 | 0 |
| 47 | [2.0, 2.5km) | E | 0 | 0 |
| 48 | [2.0, 2.5km) | NE | 0 | 0 |
